# The genome sequence of the Hoary Footman, *Eilema caniola *(Hübner, 1808)

**DOI:** 10.12688/wellcomeopenres.20574.1

**Published:** 2024-01-08

**Authors:** David C. Lees, Natural History Museum Genome Acquisition Lab, Darwin Tree of Life Barcoding collective, Wellcome Sanger Institute Tree of Life Management, Samples and Laboratory team, Wellcome Sanger Institute Scientific Operations: Sequencing Operations, Wellcome Sanger Institute Tree of Life Core Informatics team, Tree of Life Core Informatics collective, Darwin Tree of Life Consortium

**Affiliations:** 1 Natural History Museum, London, England, UK

**Keywords:** Eilema caniola, Hoary Footman, genome sequence, chromosomal, Lepidoptera

## Abstract

We present a genome assembly from one female *Eilema caniola* (the Hoary Footman; Arthropoda; Insecta; Lepidoptera; Erebidae). The genome sequence is 781.7 megabases in span. Most of the assembly is scaffolded into 31 chromosomal pseudomolecules, including the W and Z sex chromosomes. The mitochondrial genome has also been assembled and is 15.42 kilobases in length. Gene annotation of this assembly on Ensembl identified 22,953 protein coding genes.

## Species taxonomy

Eukaryota; Metazoa; Eumetazoa; Bilateria; Protostomia; Ecdysozoa; Panarthropoda; Arthropoda; Mandibulata; Pancrustacea; Hexapoda; Insecta; Dicondylia; Pterygota; Neoptera; Endopterygota; Amphiesmenoptera; Lepidoptera; Glossata; Neolepidoptera; Heteroneura; Ditrysia; Obtectomera; Noctuoidea; Erebidae; Arctiinae; Lithosiini; *Eilema*; *Eilema caniola* (Hübner, 1808) (NCBI:txid987929).

## Background

The Hoary Footman, *Eilema caniola*, is a moth in the arctiine tribe Lithosiini, with a wingspan of 26–36 mm (male) and 25–32 mm (female) ( Macià *et al.*, 2022), forewing 15–17 mm ( Waring *et al.*, 2017). Its forewings are silky pale grey with an ochreous costa and the head is yellow-orange. The forewings lack underside androconia ( Macià *et al.*, 2022). The hindwings are also whiter than the very similar *E. complana* (Hübner, 1808); like it, the moth rolls its wings at rest. Although difficult to identify superficially, the male and female genitalia are distinctive ( Macià *et al*., 2022: 12–13, Figs. 49–50). The adult moth flies in the UK from late June to early August ( Randle *et al.*, 2019). However, Macià *et al.* (2022) characterise the species in Europe as bivoltine or even trivoltine, with a continuous flight period from May to October, sometimes November in Europe.

The Hoary Footman has a preference for cliffs, quarries and shingle in the UK ( Waring *et al.*, 2017), preferring xerothermic environments up to 1800 m elevation in Europe ( Macià *et al*., 2022). The larva feeds from September to late June on lichens and algae on rocks and artificial substrates as well as on the leaves of legumes such as trefoils and Kidney Vetch *Anthyllis vulneraria* ( Henwood *et al.*, 2020). The full-grown larva is over 20 mm long (see Macià *et al.*, 2022: Figs. 75-76, Fig. 94). Pupation is on the ground in a loose cocoon.

*Eilema caniola* occurs in England, historically occurring in its southwest, western Wales, Anglesey, Scilly Isles and in the Channel Isles ( Waring *et al.*, 2017). It also occurs in southeast England as a migrant and since 2000 has spread rapidly inland; it is locally common in England and Wales and near threatened in Ireland, occurring on its southeastern coast ( Randle *et al.*, 2019). In Western Europe it is widely distributed, ranging from the shores of the Mediterranean as far north as Denmark, and also eastwards to the borders of the Black Sea ( GBIF Secretariat, 2023). Macià *et al*. (2022) describe the species as holomediterranean, occurring also in North Africa, Asia Minor and large Mediterranean islands.

Macià *et al*. (2022) propose the genus *Eilema* Hübner, [1808] as monotypic, including only *E. caniola* ( *Bombyx caniola* Hübner, 1808 is the type species). The species exhibits a single mitochondrial cluster on BOLD, the BIN BOLD:AAF6264 (30/10/2023). The DNA barcode of the individual from south-eastern Kent on which the genome is based does not differ from the most prevalent continental haplotype. Macià *et al.* (2022) recognise an additional subspecies, *E. c. torstenii* Mentzer, 1980, as endemic to the Balearics, although thus identified individuals are (30/10/2023) part of two subclusters (one of which includes individuals currently identified on BOLD as *E. complana* from Corsica; a second subcluster from there are mostly (mis-) identified as *E. complana*). The correct BIN cluster for *E. complana* is BOLD:AAB6846 (which is shared also with *E. pseudocomplana* Daniel, 1939 and *E. iberica* Mentzer, 1980; Ortiz *et al.,* 2017), which considering UK sequences on BOLD, is about 5.2% divergent to *E. caniola* (BOLD:AAF6264). In a tree based on COX1, ArgK and DDX23, the sister clade of *E. caniola* was supported as the clade *E. palliatella* (Scopoli, 1763) + *E. costalis* (Zeller, 1847) + *E. pseudocomplana* + ( *E. complana* + *E. iberica*), all of which were placed in the genus *Manulea* Wallengren, 1863 ( Macià *et al.*, 2022). The genome will be useful in helping to resolve or confirm such phylogenetic relationships, if not overall classification of *Eilema* (s.l.), which is highly polyphyletic in the latest scheme ( Macià *et al.*, 2022).

The genome of the Hoary Footman, *Eilema caniola*, was sequenced as part of the Darwin Tree of Life Project, a collaborative effort to sequence all named eukaryotic species in the Atlantic Archipelago of Britain and Ireland. Here we present a chromosomally complete genome sequence for *Eilema caniola*, based on one female specimen from Sandwich Bay, SE Kent.

## Genome sequence report

**Figure 1.**
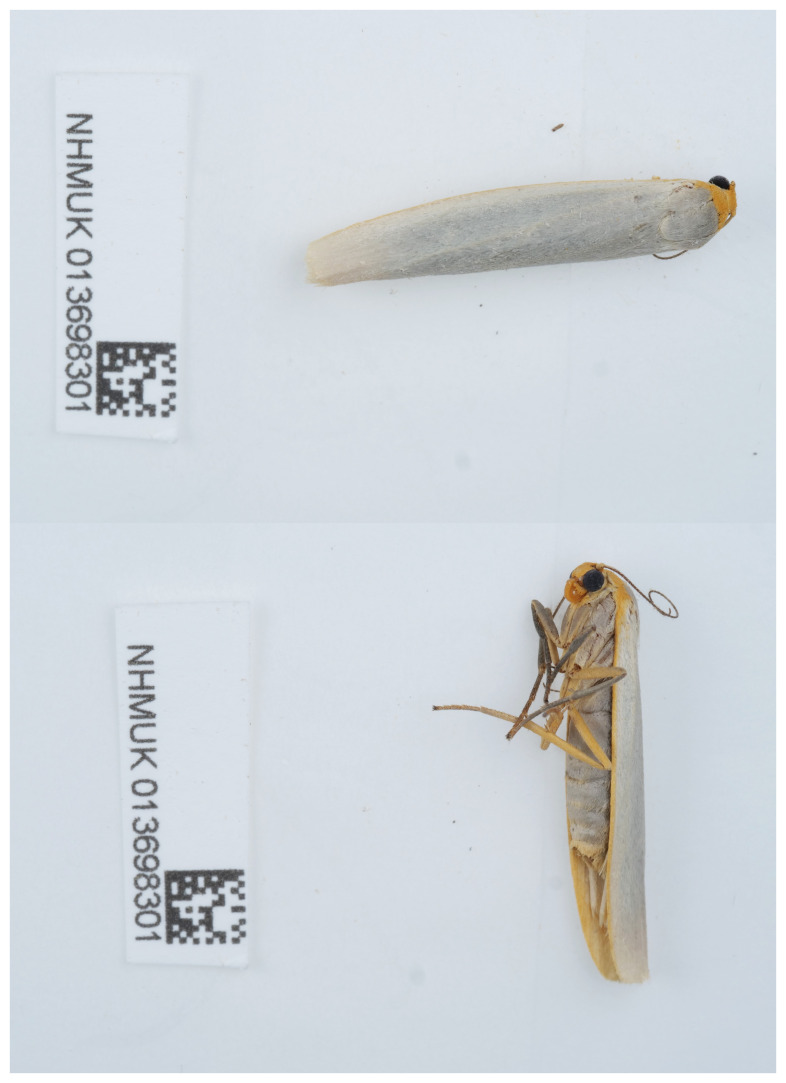
Photograph of the *Eilema caniola* (ilEilCani1) specimen used for genome sequencing.

The genome was sequenced from one female *Eilema caniola* ( Figure 1) (see Methods). A total of 39-fold coverage in Pacific Biosciences single-molecule HiFi long reads was generated. Primary assembly contigs were scaffolded with chromosome conformation Hi-C data. Manual assembly curation corrected 21 missing joins or mis-joins and removed 4 haplotypic duplications, reducing the scaffold number by 7.14%.

**Figure 2.**
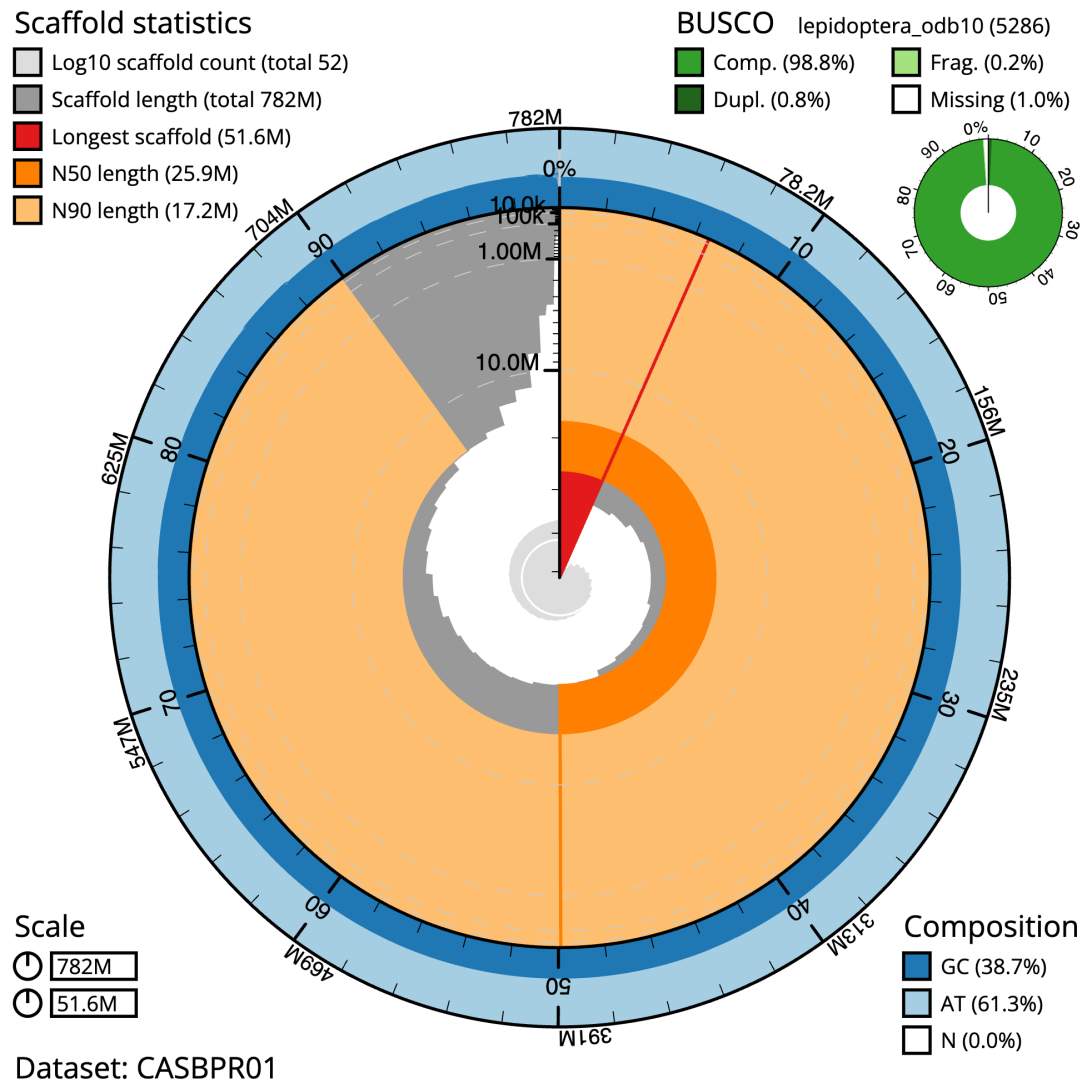
Genome assembly of *Eilema caniola*, ilEilCani1.1: metrics. The BlobToolKit Snailplot shows N50 metrics and BUSCO gene completeness. The main plot is divided into 1,000 size-ordered bins around the circumference with each bin representing 0.1% of the 781,750,262 bp assembly. The distribution of scaffold lengths is shown in dark grey with the plot radius scaled to the longest scaffold present in the assembly (51,636,824 bp, shown in red). Orange and pale-orange arcs show the N50 and N90 scaffold lengths (25,911,362 and 17,159,098 bp), respectively. The pale grey spiral shows the cumulative scaffold count on a log scale with white scale lines showing successive orders of magnitude. The blue and pale-blue area around the outside of the plot shows the distribution of GC, AT and N percentages in the same bins as the inner plot. A summary of complete, fragmented, duplicated and missing BUSCO genes in the lepidoptera_odb10 set is shown in the top right. An interactive version of this figure is available at https://blobtoolkit.genomehubs.org/view/Eilema%20caniola/dataset/CASBPR01/snail.

**Figure 3.**
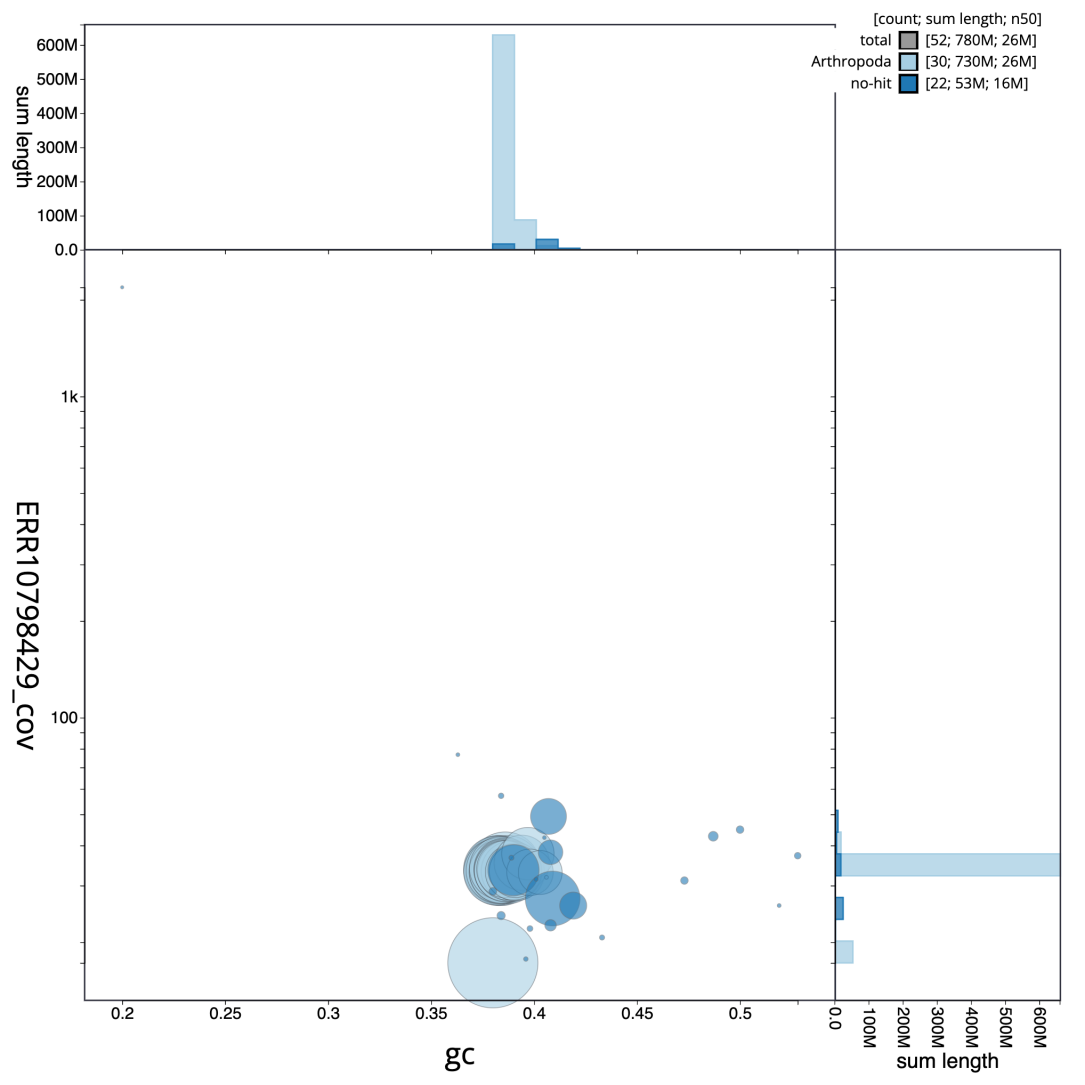
Genome assembly of *Eilema caniola*, ilEilCani1.1: BlobToolKit GC-coverage plot. Scaffolds are coloured by phylum. Circles are sized in proportion to scaffold length. Histograms show the distribution of scaffold length sum along each axis. An interactive version of this figure is available at https://blobtoolkit.genomehubs.org/view/Eilema%20caniola/dataset/CASBPR01/blob.

**Figure 4.**
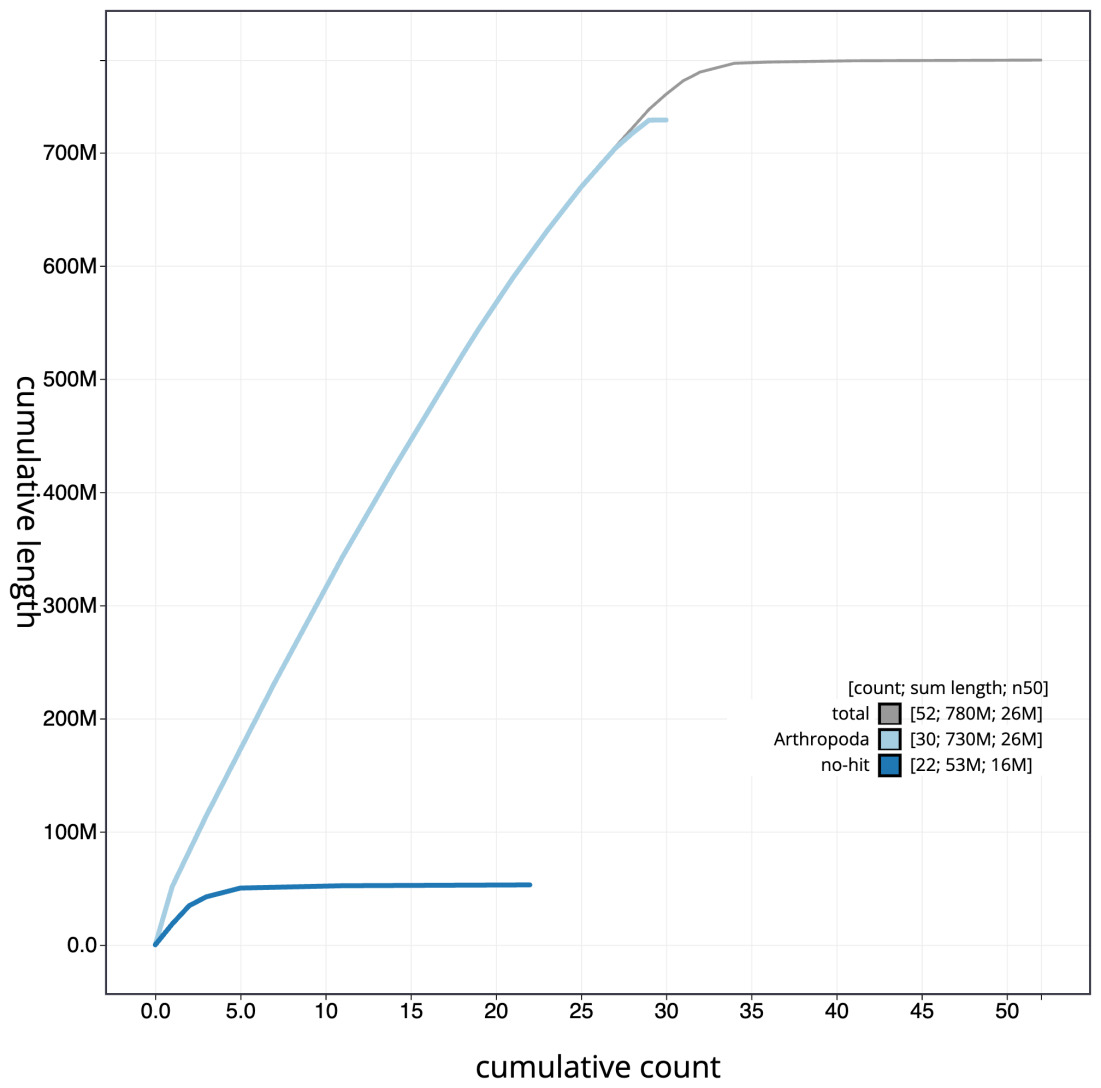
Genome assembly of *Eilema caniola*, ilEilCani1.1: BlobToolKit cumulative sequence plot. The grey line shows cumulative length for all scaffolds. Coloured lines show cumulative lengths of scaffolds assigned to each phylum using the buscogenes taxrule. An interactive version of this figure is available at https://blobtoolkit.genomehubs.org/view/Eilema%20caniola/dataset/CASBPR01/cumulative.

**Figure 5.**
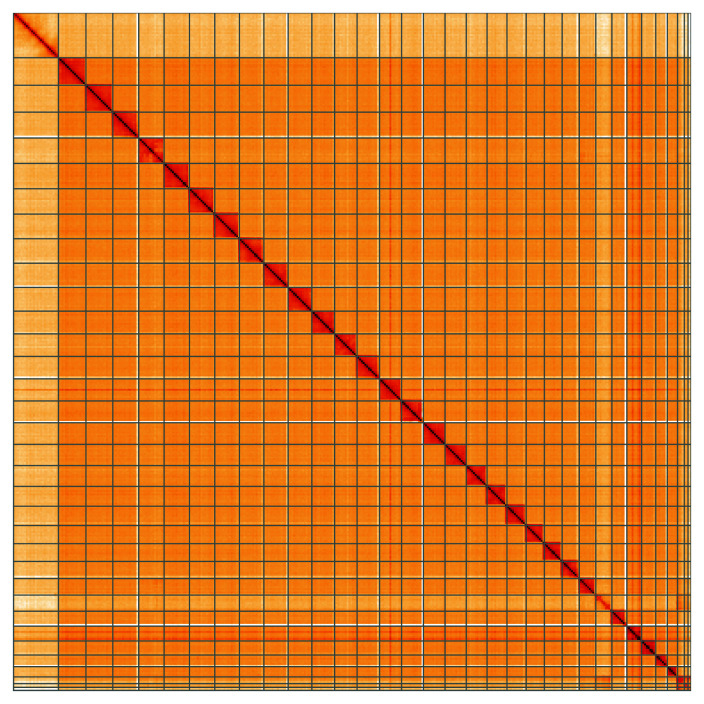
Genome assembly of *Eilema caniola*, ilEilCani1.1: Hi-C contact map of the ilEilCani1.1 assembly, visualised using HiGlass. Chromosomes are shown in order of size from left to right and top to bottom. An interactive version of this figure may be viewed at https://genome-note-higlass.tol.sanger.ac.uk/l/?d=cu-2fCbuQ1yiZr4aQHR1pQ.

The final assembly has a total length of 781.7 Mb in 51 sequence scaffolds with a scaffold N50 of 25.9 Mb ( Table 1). The snailplot in Figure 2 provides a summary of the assembly statistics, while the distribution of assembly scaffolds on GC proportion and coverage is shown in Figure 3. The cumulative assembly plot in Figure 4 shows curves for subsets of scaffolds assigned to different phyla. Most (99.71%) of the assembly sequence was assigned to 31 chromosomal-level scaffolds, representing 29 autosomes and the W and Z sex chromosomes. The order and orientation of the W chromosome is or uncertain order and orientation. Chromosome-scale scaffolds confirmed by the Hi-C data are named in order of size ( Figure 5; Table 2). While not fully phased, the assembly deposited is of one haplotype. Contigs corresponding to the second haplotype have also been deposited. The mitochondrial genome was also assembled and can be found as a contig within the multifasta file of the genome submission.

**Table 1.** Genome data for *Eilema caniola*, ilEilCani1.1.

Project accession data		
Assembly identifier	ilEilCani1.1	
Species	*Eilema caniola*	
Specimen	ilEilCani1	
NCBI taxonomy ID	987929	
BioProject	PRJEB58964	
BioSample ID	SAMEA111458562	
Isolate information	ilEilCani1, female: head and thorax (DNA sequencing and Hi-C scaffolding)	
Assembly metrics *		*Benchmark*
Consensus quality (QV)	65.1	*≥ 50*
*k*-mer completeness	100%	*≥ 95%*
BUSCO **	C:98.8%[S:98.1%,D:0.8%],F:0.2%,M:1.0%,n:5,286	*C ≥ 95%*
Percentage of assembly mapped to chromosomes	99.71%	*≥ 95%*
Sex chromosomes	W and Z chromosomes	*localised homologous pairs*
Organelles	Mitochondrial genome assembled	*complete single alleles*
Raw data accessions		
PacificBiosciences SEQUEL II	ERR10798429	
Hi-C Illumina	ERR10786033	
Genome assembly		
Assembly accession	GCA_949126895.1	
*Accession of alternate haplotype*	GCA_949126945.1	
Span (Mb)	781.7	
Number of contigs	135	
Contig N50 length (Mb)	9.9	
Number of scaffolds	51	
Scaffold N50 length (Mb)	25.9	
Longest scaffold (Mb)	51.5	
Genome annotation		
Number of protein-coding genes	22,953	
Number of gene transcripts	23,146	

* Assembly metric benchmarks are adapted from column VGP-2020 of “Table 1: Proposed standards and metrics for defining genome assembly quality” from ( Rhie *et al.*, 2021). ** BUSCO scores based on the lepidoptera_odb10 BUSCO set using v5.3.2. C = complete [S = single copy, D = duplicated], F = fragmented, M = missing, n = number of orthologues in comparison. A full set of BUSCO scores is available at https://blobtoolkit.genomehubs.org/view/Eilema%20caniola/dataset/CASBPR01/busco.

**Table 2.** Chromosomal pseudomolecules in the genome assembly of *Eilema caniola*, ilEilCani1.

INSDC accession	Chromosome	Length (Mb)	GC%
OX421462.1	1	31.63	38.5
OX421463.1	2	30.9	38.5
OX421464.1	3	29.74	38.5
OX421465.1	4	29.22	38.5
OX421466.1	5	29.13	38.5
OX421467.1	6	29.09	38.5
OX421468.1	7	28.39	38.5
OX421469.1	8	28.09	38.0
OX421470.1	9	27.85	38.0
OX421471.1	10	27.27	38.5
OX421472.1	11	26.13	38.5
OX421473.1	12	25.91	38.5
OX421474.1	13	25.83	38.5
OX421475.1	14	25.33	38.5
OX421476.1	15	25.1	38.5
OX421477.1	16	24.84	38.5
OX421478.1	17	24.41	38.5
OX421479.1	18	23.9	38.5
OX421480.1	19	22.86	39.0
OX421481.1	20	22.12	38.5
OX421482.1	21	20.89	39.0
OX421483.1	22	20.42	38.5
OX421484.1	23	19.72	39.0
OX421485.1	24	18.99	39.5
OX421486.1	25	17.16	39.0
OX421487.1	26	17.02	39.5
OX421488.1	27	16.18	39.0
OX421489.1	28	13.32	40.0
OX421490.1	29	11.79	40.5
OX421461.1	W	18.54	41.0
OX421460.1	Z	51.64	38.0
OX421491.1	MT	0.02	20.0

The estimated Quality Value (QV) of the final assembly is 65.1 with *k*-mer completeness of 100%, and the assembly has a BUSCO v5.3.2 completeness of 98.8% (single = 98.1%, duplicated = 0.8%), using the lepidoptera_odb10 reference set ( *n* = 5,286).

Metadata for specimens, barcode results, spectra estimates, sequencing runs, contaminants and pre-curation assembly statistics are given at https://links.tol.sanger.ac.uk/species/987929.

## Genome annotation report

The *Eilema caniola* genome assembly (GCA_949126895.1) was annotated using the Ensembl rapid annotation pipeline ( Table 1; https://rapid.ensembl.org/Eilema_caniola_GCA_949126895.1/Info/Index). The resulting annotation includes 23,146 transcribed mRNAs from 22,953 protein-coding genes.

## Methods

### Sample acquisition and nucleic acid extraction

A female *Eilema caniola* (specimen ID NHMUK013698301, ToLID ilEilCani1) was collected by hand from Restharrow Dunes National Nature Reserve, Sandwich Bay, England, UK (latitude 51.27, longitude 1.38) on 2021-09-24. The specimen was collected and identified by David Lees (Natural History Museum) and preserved by dry freezing at –80°C.

The workflow for high molecular weight (HMW) DNA extraction at the Wellcome Sanger Institute (WSI) includes a sequence of core procedures: sample preparation; sample homogenisation; DNA extraction; HMW DNA fragmentation; and fragmented DNA clean-up. The sample was prepared for DNA extraction at the WSI Tree of Life laboratory: the ilAnaCroc1 sample was weighed and dissected on dry ice with tissue set aside for Hi-C sequencing ( Jay *et al.*, 2023). Tissue from the whole organism was homogenised using a PowerMasher II tissue disruptor ( Denton *et al.*, 2023a). HMW DNA was extracted in the WSI Scientific Operations core using the Automated MagAttract v2 protocol ( Oatley *et al.*, 2023). The DNA was sheared into an average fragment size of 12–20 kb in a Megaruptor 3 system with speed setting 31 ( Bates *et al.*, 2023). Sheared DNA was purified by solid-phase reversible immobilisation ( Strickland *et al.*, 2023): in brief, the method employs a 1.8X ratio of AMPure PB beads to sample to eliminate shorter fragments and concentrate the DNA. The concentration of the sheared and purified DNA was assessed using a Nanodrop spectrophotometer and Qubit Fluorometer and Qubit dsDNA High Sensitivity Assay kit. Fragment size distribution was evaluated by running the sample on the FemtoPulse system.

Protocols developed in the Tree of Life laboratory are publicly available on protocols.io ( Denton *et al.*, 2023b).

### Sequencing

Pacific Biosciences HiFi circular consensus DNA sequencing libraries were constructed according to the manufacturers’ instructions. DNA sequencing was performed by the Scientific Operations core at the WSI on a Pacific Biosciences SEQUEL II instrument. Hi-C data were also generated from remaining head and thorax tissue of ilEilCani1 using the Arima2 kit and sequenced on the Illumina NovaSeq 6000 instrument.

### Genome assembly, curation and evaluation

Assembly was carried out with Hifiasm ( Cheng *et al.*, 2021) and haplotypic duplication was identified and removed with purge_dups ( Guan *et al.*, 2020). The assembly was then scaffolded with Hi-C data ( Rao *et al.*, 2014) using YaHS ( Zhou *et al.*, 2023). The assembly was checked for contamination and corrected as described previously ( Howe *et al.*, 2021). Manual curation was performed using HiGlass ( Kerpedjiev *et al.*, 2018) and Pretext ( Harry, 2022). The mitochondrial genome was assembled using MitoHiFi ( Uliano-Silva *et al.*, 2023), which runs MitoFinder ( Allio *et al.*, 2020) or MITOS ( Bernt *et al.*, 2013) and uses these annotations to select the final mitochondrial contig and to ensure the general quality of the sequence.

A Hi-C map for the final assembly was produced using bwa-mem2 ( Vasimuddin *et al.*, 2019) in the Cooler file format ( Abdennur & Mirny, 2020). To assess the assembly metrics, the *k*-mer completeness and QV consensus quality values were calculated in Merqury ( Rhie *et al.*, 2020). This work was done using Nextflow ( Di Tommaso *et al.*, 2017) DSL2 pipelines “sanger-tol/readmapping” ( Surana *et al.*, 2023a) and “sanger-tol/genomenote” ( Surana *et al.*, 2023b). The genome was analysed within the BlobToolKit environment ( Challis *et al.*, 2020) and BUSCO scores ( Manni *et al.*, 2021; Simão *et al.*, 2015) were calculated.

Table 3 contains a list of relevant software tool versions and sources.

**Table 3.** Software tools: versions and sources.

Software tool	Version	Source
BlobToolKit	4.1.5	https://github.com/blobtoolkit/blobtoolkit
BUSCO	5.3.2	https://gitlab.com/ezlab/busco
Hifiasm	0.16.1-r375	https://github.com/chhylp123/hifiasm
HiGlass	1.11.6	https://github.com/higlass/higlass
Merqury	MerquryFK	https://github.com/thegenemyers/MERQURY.FK
MitoHiFi	2	https://github.com/marcelauliano/MitoHiFi
PretextView	0.2	https://github.com/wtsi-hpag/PretextView
purge_dups	1.2.3	https://github.com/dfguan/purge_dups
sanger-tol/genomenote	v1.0	https://github.com/sanger-tol/genomenote
sanger-tol/readmapping	1.1.0	https://github.com/sanger-tol/readmapping/tree/1.1.0
YaHS	1.2a	https://github.com/c-zhou/yahs

### Genome annotation

The BRAKER2 pipeline ( Brůna *et al.*, 2021) was used in the default protein mode to generate annotation for the *Eilema caniola* assembly (GCA_949126895.1) in Ensembl Rapid Release.

### Wellcome Sanger Institute – Legal and Governance

The materials that have contributed to this genome note have been supplied by a Darwin Tree of Life Partner. The submission of materials by a Darwin Tree of Life Partner is subject to the **‘Darwin Tree of Life Project Sampling Code of Practice’**, which can be found in full on the Darwin Tree of Life website here. By agreeing with and signing up to the Sampling Code of Practice, the Darwin Tree of Life Partner agrees they will meet the legal and ethical requirements and standards set out within this document in respect of all samples acquired for, and supplied to, the Darwin Tree of Life Project.

Further, the Wellcome Sanger Institute employs a process whereby due diligence is carried out proportionate to the nature of the materials themselves, and the circumstances under which they have been/are to be collected and provided for use. The purpose of this is to address and mitigate any potential legal and/or ethical implications of receipt and use of the materials as part of the research project, and to ensure that in doing so we align with best practice wherever possible. The overarching areas of consideration are:

•   Ethical review of provenance and sourcing of the material

•   Legality of collection, transfer and use (national and international)

Each transfer of samples is further undertaken according to a Research Collaboration Agreement or Material Transfer Agreement entered into by the Darwin Tree of Life Partner, Genome Research Limited (operating as the Wellcome Sanger Institute), and in some circumstances other Darwin Tree of Life collaborators.

## Data Availability

European Nucleotide Archive: *Eilema caniola*. Accession number PRJEB58964; https://identifiers.org/ena.embl/PRJEB58964 ( Wellcome Sanger Institute, 2023). The genome sequence is released openly for reuse. The *Eilema caniola* genome sequencing initiative is part of the Darwin Tree of Life (DToL) project. All raw sequence data and the assembly have been deposited in INSDC databases. Raw data and assembly accession identifiers are reported in Table 1.

## Author information

Members of the Natural History Museum Genome Acquisition Lab are listed here: https://doi.org/10.5281/zenodo.7139035.

Members of the Darwin Tree of Life Barcoding collective are listed here: https://doi.org/10.5281/zenodo.4893703.

Members of the Wellcome Sanger Institute Tree of Life Management, Samples and Laboratory team are listed here: https://doi.org/10.5281/zenodo.10066175.

Members of Wellcome Sanger Institute Scientific Operations: Sequencing Operations are listed here: https://doi.org/10.5281/zenodo.10043364.

Members of the Wellcome Sanger Institute Tree of Life Core Informatics team are listed here: https://doi.org/10.5281/zenodo.10066637.

Members of the Tree of Life Core Informatics collective are listed here: https://doi.org/10.5281/zenodo.5013541.

Members of the Darwin Tree of Life Consortium are listed here: https://doi.org/10.5281/zenodo.4783558.
