# Supplementary material for: The genome sequence of the Hoary Footman, Eilema caniola (Hübner, 1808)
Source: Wellcome Open Res. 2024 Jan 8;9:5. [Version 1] doi: 10.12688/wellcomeopenres.20574.1 (PMC11109690; doi:10.12688/wellcomeopenres.20574.1)
Supplement: Peer review history

# Reviewer report: Reviewer response for version 1

**DOI:** 10.21956/wellcomeopenres.22773.r77792

**Published:** 2024-05-10

**Authors:** Arun Arumugaperumal

**Affiliations:** 1 Department of Biotechnology, Rajalakshmi Engineering College, Chennai, Tamilnadu, India

The paper presents the whole genome of Eilema caniola known as the Hoary Footman. This is the first report of the high quality whole genome data of the insect. The insect genome was found to be of size 781.7 Mbp. The genome sequence was scaffolded into 31 chromosomes. The contig containing the mitogenome was identified and found to be of size 15.4 kbp. 22,953 genes were identified in the genome. The genes were well annotated.

 The author had used Hi-C data to scaffold the contigs. The scaffold N50 length was 25.9 Mb. This indicates a well assembled genome. BUSCO analyis has shown that the genome sequence is 98.8% complete. The non-coding RNA data can be accessed from the annotations ontained through Ensembl.

 The data generated in this article were deposited in public databases. The link to access the datasets and annotations were provided in the manuscript.

Are sufficient details of methods and materials provided to allow replication by others?

Yes

Is the rationale for creating the dataset(s) clearly described?

Yes

Are the datasets clearly presented in a useable and accessible format?

Yes

Are the protocols appropriate and is the work technically sound?

Yes

Reviewer Expertise:

Genomics, Bioinformatics

I confirm that I have read this submission and believe that I have an appropriate level of expertise to confirm that it is of an acceptable scientific standard.

# Reviewer report: Reviewer response for version 1

**DOI:** 10.21956/wellcomeopenres.22773.r77788

**Published:** 2024-04-05

**Authors:** Chiara Bortoluzzi

**Affiliations:** 1 Department of Environmental Systems Science, ETH Zurich (Ringgold ID: 27219), Zürich, Zürich, Switzerland

In this genome note, the Darwin Tree of Life consortium report the sequencing of the Hoary Footman ( *Eilema caniola*) genome, a species of moth endemic to the United Kingdom. This genome is a high quality one and will play a significant role in resolving or confirming the evolutionary relationships of Eilema caniola.

 The report is very well written and datasets are presented in a clear manner. The data is readily accessible to the public through both ENA and NCBI, with all provided links functioning properly. The values presented in tables are accurate and align with the information available in public repositories.

 I suggest the following changes to the main text:

**Page 3/11:**

1. Replace "Kidney Vetch" with "kidney vetch"
2. Replace "Although thus identified individuals" with "Although these identified individuals"
3. Replace “if not overall classification” with "if not the overall classification"
4. Replace "SE Kent" with "south-east Ken"

**Page 5/11:**

 Replace "The order and orientation of the W chromosome is or uncertain order and orientation" with "The order and orientation of the W chromosome is of uncertain order and orientation"

**Page 7/11:**

 Replace "ilAnaCroc1" with "ilEilCani1"

 Change the orientation of the label on the y-axis of Figure 3 and Figure 4 to improve readability.

Are sufficient details of methods and materials provided to allow replication by others?

Yes

Is the rationale for creating the dataset(s) clearly described?

Yes

Are the datasets clearly presented in a useable and accessible format?

Yes

Are the protocols appropriate and is the work technically sound?

Yes

Reviewer Expertise:

Comparative genomics,  population genomics, conservation genomics

I confirm that I have read this submission and believe that I have an appropriate level of expertise to confirm that it is of an acceptable scientific standard.
